# Supplementing Consent for a Prospective Longitudinal Cohort Study of Infants With Antenatal Opioid Exposure: Development and Assessment of a Digital Tool

**DOI:** 10.2196/59954

**Published:** 2025-03-04

**Authors:** Jamie E Newman, Leslie Clarke, Pranav Athimuthu, Megan Dhawan, Sharon Owen, Traci Beiersdorfer, Lindsay M Parlberg, Ananta Bangdiwala, Taya McMillan, Sara B DeMauro, Scott Lorch, Myriam Peralta-Carcelen, Deanne Wilson-Costello, Namasivayam Ambalavanan, Stephanie L Merhar, Brenda Poindexter, Catherine Limperopoulos, Jonathan M Davis, Michele Walsh, Carla M Bann

**Affiliations:** 1 RTI International, 3040 Cornwallis Road, Research Triangle Park, NC, 27709, United States, 1 9194855719; 2 Case Western Reserve University, Cleveland, OH, United States; 3 Children's Hospital of Philadelphia, Philadelphia, PA, United States; 4 University of Alabama at Birmingham, Birmingham, AL, United States; 5 University of Cincinnati College of Medicine, Cincinnati Children's Hospital Medical Center, Cincinnati, OH, United States; 6 Emory University, Atlanta, GA, United States; 7 Children's National Hospital, Washington, DC, United States; 8 Tufts Medical Center, Boston, MA, United States; 9 National Institute of Child Health and Human Development, Rockville, MD, United States

**Keywords:** informed consent digital tool, avatars, video-assisted consent, MRI, antenatal opioid exposure, infant, antenatal, opioid exposure, caregiver, survey, magnetic resonance imaging, Outcomes of Babies With Opioid Exposure

## Abstract

**Background:**

The Outcomes of Babies With Opioid Exposure (OBOE) study is an observational cohort study examining the impact of antenatal opioid exposure on outcomes from birth to 2 years of age. COVID-19 social distancing measures presented challenges to research coordinators discussing the study at length with potential participants during the birth hospitalization, which impacted recruitment, particularly among caregivers of unexposed (control) infants. In response, the OBOE study developed a digital tool (consenter video) to supplement the informed consent process, make it more engaging, and foster greater identification with the research procedures among potential participants.

**Objective:**

We aim to examine knowledge of the study, experiences with the consent process, and perceptions of the consenter video among potential participants of the OBOE study.

**Methods:**

Analyses included 129 caregivers who were given the option to view the consenter video as a supplement to the consent process. Participants selected from 3 racially and ethnically diverse avatars to guide them through the 11-minute video with recorded voice-overs. After viewing the consenter video, participants completed a short survey to assess their knowledge of the study, experiences with the consent process, and perceptions of the tool, regardless of their decision to enroll in the main study. Chi-square tests were used to assess differences between caregivers of opioid-exposed and unexposed infants in survey responses and whether caregivers who selected avatars consistent with their racial or ethnic background were more likely to enroll in the study than those who selected avatars that were not consistent with their background.

**Results:**

Participants demonstrated good understanding of the information presented, with 95% (n=123) correctly identifying the study purpose and 88% (n=112) correctly indicating that their infant would not be exposed to radiation during the magnetic resonance imaging. Nearly all indicated they were provided “just the right amount of information” (n=123, 98%) and that they understood the consent information well enough to decide whether to enroll (n=125, 97%). Survey responses were similar between caregivers of opioid-exposed infants and unexposed infants on all items except the decision to enroll. Those in the opioid-exposed group were more likely to enroll in the main study compared to the unexposed group (n=49, 89% vs n=38, 51%; *P*<.001). Of 81 caregivers with known race or ethnicity, 35 (43%) chose avatars to guide them through the video that matched their background. Caregivers selecting avatars consistent with their racial or ethnic background were more likely to enroll in the main study (n=29, 83% vs n=43, 57%; *P*=.01).

**Conclusions:**

This interactive digital tool was helpful in informing prospective participants about the study. The consenter tool enhanced the informed consent process, reinforced why caregivers of unexposed infants were being approached, and was particularly helpful as a resource for families to understand magnetic resonance imaging procedures.

## Introduction

The importance of the informed consent process in today’s clinical trials stems from ethical standards detailed in landmark documents such as the Nuremberg Code, the Declaration of Helsinki, and the Belmont Report. Recent research suggests that there is room for improvement in the consent processes to better inform prospective participants of study aims and potential risks. Numerous studies have suggested that minority populations, those with low levels of education, and those with intellectual disabilities are among the groups in which improvements could be made to heighten participants’ understanding of the informed consent process (see Tam et al [1] for a review). Some studies have observed that fewer than half of the participants were able to correctly answer questions about the study’s purpose after reviewing an informed consent form [2,3]. In the review by Tam et al [1], only 55% could name a risk associated with a study, with even fewer being able to define key methodological concepts such as the use of placebo or randomization.

Informed consent materials should be easily comprehended by the average research participant. Grade-level reading standards are the most common metric for measuring the readability of consent documents. It is generally agreed that material should target 6th- to 8th-grade level standards to be considered widely interpretable. Yet, a 2003 study exploring informed consent materials furnished by research institutions across the United States found that only 8% of their sample met their own institution’s standards for comprehension, with the average readability score of a consent document exceeding stated standards by almost 3 grade levels [4]. If prospective participants cannot understand the structure and content of a consent form, they may decline to participate. If a study using English language materials involves nonnative English speakers, minority populations, or foreign immigrants, the reading level must be further simplified [5].

In response to criticisms of written consent forms, recent studies have harnessed technology to create consent procedures that simplify the information being conveyed while better engaging prospective participants. While some approaches have used electronic forms, others have developed videos, audio files, and multimedia tutorials to supplement the consent process.

Electronic consent tools found their earliest uses in medical procedures, and most evaluations of their effectiveness continue to reside in that domain [6,7]. Studies evaluating electronic consent have focused on comprehension [8], engagement [8-10], satisfaction and procedure-related anxiety reduction [11], recall of risks and benefits [4], and the potential to streamline the consent process by reducing time spent by staff explaining study procedures [7]. There are mixed findings on the effectiveness of video-assisted consent compared with written consent forms [9]. This may be due, in part, to technology advances over time. Videos created 20 years ago would have been limited in the graphics capacity of procedures they could display, whereas modern tools have the capacity to include 3D animation and guided walk-throughs. Multiple studies that have successfully demonstrated the effectiveness of video-assisted consent involve minority populations, study participants from rural areas, populations with English as a second language, older adults, and those with intellectual disabilities [12-15].

Certain capacities show particular promise at recruiting people traditionally underrepresented in medical research and vulnerable populations, warranting consideration in future studies. For example, Lawrence et al [16] describe an electronic consent framework using REDCap (Research Electronic Data Capture; Vanderbilt University) and customizable avatars. Videos provided participants with a choice of virtual assistants voiced by community members that guided them through the consent process. Participants had the ability to select their avatar from a variety of backgrounds, which were developed in consultation with targeted communities. Participants “emphasized the importance of representing minority populations [and] depicting supportive and helpful interactions with medical staff,” as well as their strong preference for seeing procedures depicted using avatars [16]. Participants reported being inspired by the avatars and being able to see themselves in the avatars, which have translated into stronger intervention outcomes for the treatment group [17].

Digitally assisted consent also offers potential advantages in other research domains for informing participants of study procedures and potential risks such as neuroimaging methods (eg, magnetic resonance imaging [MRI]). Although MRI is largely a low-risk, noninvasive procedure that substitutes magnetic fields for high-energy radiation, misconceptions about its use abound, including false beliefs that MRI exposes participants to radiation [18]. To our knowledge, no studies of electronic consent tools have assessed the potential of video-assisted consent to help communicate risks associated with MRI procedures. The few studies involving neuroimaging have focused on computed tomography and other radiation methods that do not carry as many misconceptions [13]. Thus, our research fills a gap in this literature by assessing whether using a video supplement to visually depict MRI scanning procedures and associated risks can adequately inform potential participants and dispel common MRI misperceptions in the Outcomes of Babies With Opioid Exposure (OBOE) study. It also builds on past literature on consent procedures, by including ethnically diverse avatars to describe MRI procedures.

Social distancing measures to mitigate the spread of COVID-19 presented new challenges to research coordinators talking at length with caregivers during the birth hospitalization about the OBOE study and building rapport through these face-to-face interactions. To mitigate these challenges, we developed the OBOE study consenter video to supplement the informed consent process and to make it more engaging for potential study participants, especially those from underrepresented populations. We developed this interactive digital tool to inform prospective participants about the study, reinforce why caregivers of unexposed (control) infants were being approached, provide a resource for families to understand the MRI procedures, and describe the longitudinal nature of the study while detailing visits across the 2-year study period. To enhance participant diversity, we developed 3 racially and ethnically diverse avatars, which participants could choose to guide them through the video. If potential participants were able to see themselves in the avatars, this may foster greater identification with the research procedures. This paper provides an overview of the digital tool and examines knowledge of the study, experiences with the consent process, and perceptions of the video among potential participants in the OBOE study.

## Methods

### OBOE Study Overview

The Advancing Clinical Trials in Neonatal Opioid Withdrawal OBOE study is a multisite prospective longitudinal cohort study of infants with antenatal opioid exposure and unexposed infants from birth to 2 years of age (ClinicalTrials.gov NCT04149509). The OBOE study protocol is described elsewhere in detail [19]. Briefly, the observational study aims to determine the impact of antenatal opioid exposure on brain development and neurodevelopmental outcomes over the first 2 years of life and to explore whether family, home, and community factors modify developmental trajectories. The study consists of 3 MRI visits at 0‐1 month, 6 months, and 24 months; 1 home visit at 12 months; 1 telephone interview at 18 months; and developmental testing at 12 and 24 months. The OBOE study addresses a growing health concern—opioid use and misuse—among a particularly vulnerable population: pregnant individuals and their infants. Overdose has become one of the leading causes of maternal mortality [20], mirroring the rise in misuse among pregnant individuals and the greater population throughout the opioid epidemic.

### Digital Tool Development: The Consenter Video

The first infant was enrolled into the OBOE study in August 2020, and the OBOE study began developing the consenter video a year later to assist with the slower-than-anticipated enrollment and to enhance participant diversity. The consenter tool is a patient-centered, interactive digital tool for informing prospective participants about clinical research studies and enhancing informed consent processes [21,22]. It transforms the informed consent process from the passive receipt of complex medical and scientific information to an active, engaging visual experience that can improve individuals’ understanding of clinical research processes and result in a more informed, dedicated research participant [23]. Five main steps were involved in developing the OBOE study consenter tool. First, a detailed storyboard was developed, which included verbatim audio text and a written description of proposed visuals. The audio text was primarily pulled from the single institutional review board (IRB)–approved consent form. Next, the team created illustrations and visuals to describe the study and assessments. Third, a group of digital designers developed customized avatars. Fourth, the team selected 3 actors to record voice-overs that corresponded to the race and ethnicity of each avatar. Finally, the video production team combined the multimedia elements into a single video file. The development process was guided by a communication scientist to ensure that evidence-based communication practices were incorporated. Throughout each step in the process, staff at the clinical sites, including research coordinators, nurses, physicians, and MRI staff, reviewed the products, and their feedback was incorporated. All study team members attended a virtual consenter orientation held before implementation. The OBOE study launched the IRB-approved consenter tool in June 2022, at which time approximately half of the target sample had been enrolled.

### Ethical Considerations

#### Human Subject Ethics Review Approvals

Through a single IRB at Cincinnati Children’s Hospital Medical Center, all 4 clinical sites (Cincinnati Children’s Hospital Medical Center, University of Alabama at Birmingham, Children’s Hospital of Pennsylvania, and Case Western Reserve University), the Neuroimaging Core at Children’s National Hospital, and the Data Coordinating Center at the RTI (Research Triangle Institute) received approval for human subjects research activities for the OBOE study, and informed consent was obtained for all participants. The STROBE (Strengthening the Reporting of Observational Studies in Epidemiology) reporting guidelines for observational studies were followed [24].

#### Informed Consent

Caregivers of opioid-exposed infants and unexposed infants were asked to view the consenter video in addition to talking to coordinators about the study as well as reading and signing the informed consent form. Caregivers of infants born at or after 37 weeks gestation were recruited at the 4 clinical sites primarily during hospital stays following birth and, to a lesser extent, prenatal clinics. Caregivers of opioid-exposed infants were approached if their infant was exposed to opioids in the second or third trimester of pregnancy. Caregivers of unexposed (control) infants were approached if there was no antenatal drug exposure as determined by maternal history or maternal urine toxicology screen at delivery. OBOE study exclusion criteria for both groups were confirmed at delivery and are detailed elsewhere [19].

#### Privacy and Confidentiality

**Figure 1.**
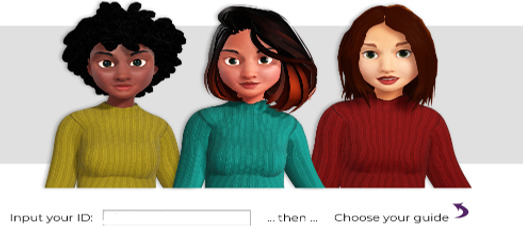
Consenter avatars that caregivers choose to guide them through the video as a supplement to the informed consent form.

Caregivers approached for the OBOE study were assigned a participant ID number, and they chose from one of three racially and ethnically diverse avatars to guide them through the 11-minute video with recorded voiceovers and illustrations of study procedures (Figure 1). After viewing the video, caregivers were asked to complete a brief survey using their participant ID, regardless of their decision to participate in the main study. The survey assessed their knowledge of the study, experiences with the consent process, and perception of the video.

#### Compensation Details

Participants were given a US $10 gift card for completing the survey.

### Digital Tool Overview

To ensure ease of access to the consenter tool, participants were provided 3 options for viewing the video. These included: (1) an iPad with the video preloaded onto an app, which the participants used alongside a coordinator; (2) a coordinator lanyard card with a QR code that participants could scan to watch the consenter video on their personal mobile device; and (3) business cards with a QR code and web link directing to the video so that participants could access the video asynchronously away from the study site.

**Figure 2.**
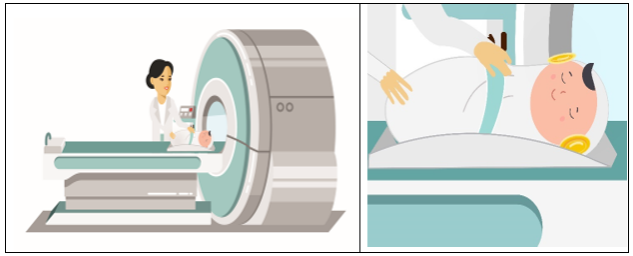
Consenter video screenshots illustrating MRI procedures at the 0‐1 month OBOE study visit. MRI: magnetic resonance imaging; OBOE: Outcomes of Babies With Opioid Exposure.

The interactive video described the study purpose, detailed the risks and benefits of joining the study, explained that participation is voluntary, and depicted MRI procedures via a verbal description and illustrations of a staff member preparing a swaddled, sleeping baby with protective earmuffs to enter the MRI scanner (Figure 2). The avatar explained that MRI scans are safe and do not expose the infant to any radiation, dispelling a common misperception of MRI.

**Figure 3.**
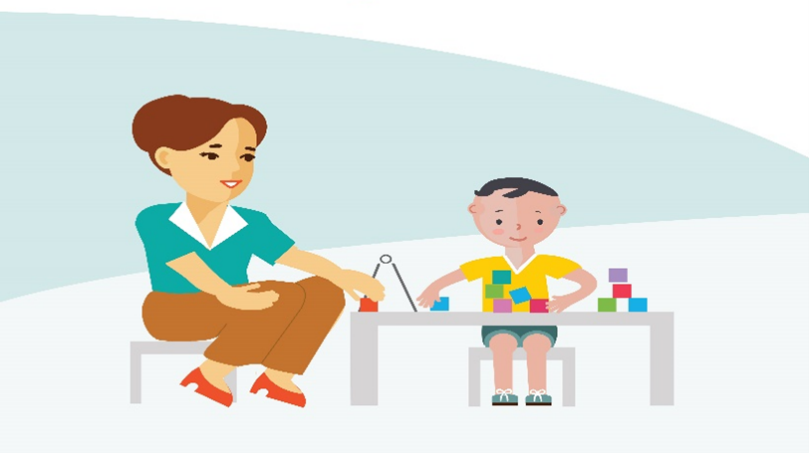
Consenter video screenshot illustrating developmental testing at the 24-month OBOE study visit. OBOE: Outcomes of Babies With Opioid Exposure.

The consenter tool detailed the longitudinal nature of the study and described assessments at each visit time point. Figure 3 illustrates developmental testing at the 24-month visit.

### Statistical Analysis

All analyses were performed using SAS (version 9.4; SAS Institute). Chi-square tests were used to examine (1) whether there were differences between opioid-exposed and unexposed groups in survey responses and (2) whether participants who selected avatars consistent with their racial or ethnic background were more likely to report that they decided to enroll in the study than those who selected avatars that were not consistent with their background.

## Results

### Overview

Analyses included 129 caregivers (55 caregivers of opioid-exposed infants and 74 caregivers of unexposed infants) who were given the option to view the consenter video as a supplement to the informed consent process and take the survey.

### Survey Responses

Participants demonstrated a good understanding of the information presented in the video (Table 1). Overall, 95% (n=123) correctly identified the purpose of the OBOE study. The majority also correctly indicated that the research team would collect the baby’s umbilical cord (n=120, 93%), the infant would not be exposed to radiation during the MRI scan (n=112, 88%), and the study would not include a blood test (n=105, 83%). Nearly all indicated they were provided “just the right amount of information” (n=123, 98%) and understood the consent information well enough to decide whether to enroll (n=125, 97%).

**Table 1.** Survey responses of caregivers of opioid-exposed infants and unexposed infants after watching consenter video (N=129).

Item	All (n=129), n (%)	Opioid exposed (n=55), n (%)	Unexposed (n=74), n (%)
Knowledge of study			
1. Correctly identified the purpose of the OBOE^a^ study	123 (95)	53 (96)	70 (95)
2. Correctly indicated, “If I enroll in the study, the research team will collect a piece of my baby’s umbilical cord at delivery that would otherwise be thrown away.”	120 (93)	50 (91)	70 (95)
3. Correctly indicated that infant would not be exposed to radiation during the MRI^b^ scan	112 (88)	48 (87)	64 (88)
4. Which of the following is not one of the study activities:			
a. Three brain MRIs over the first 2 years of my baby’s life	4 (3)	3 (5)	1 (1)
b. A 12-month home visit	9 (7)	6 (11)	3 (4)
c. A blood test (correct response)	105 (83)	44 (80)	61 (86)
d. Parent or caregiver surveys	8 (6)	2 (4)	6 (8)
Experience with consent process			
5. During the consent process, do you feel you were provided:			
a. Too much information	0 (0)	0 (0)	0 (0)
b. Just the right amount of information	123 (98)	53 (98)	70 (97)
c. Too little information	3 (2)	1 (2)	2 (3)
6. Felt you understood the consent information well enough to decide whether you and your baby should be in the study	125 (97)	55 (100)	70 (95)
Perceptions of consenter video			
7. Did you watch the video describing the study?			
a. Yes	124 (96)	51 (93)	73 (99)
b. No	5 (4)	4 (7)	1 (1)
7a. If yes, what did you like most about the video^c^?			
a. The explanation of why the study is important	62 (51)	27 (53)	35 (49)
b. The explanation of the study procedures	28 (23)	12 (24)	16 (23)
c. The explanation of the MRI	19 (16)	6 (12)	13 (18)
d. The explanation of how my personal information would be protected	7 (6)	4 (8)	3 (4)
e. I did not like watching the video	2 (2)	1 (2)	1 (1)
f. Other	4 (3)	1 (2)	3 (4)
7b. If yes, what did you not like about the video?			
a. It was too long	5 (4)	1 (2)	4 (6)
b. It was hard to understand	0 (0)	0 (0)	0 (0)
c. It did not cover everything I needed to know about the study	0 (0)	0 (0)	0 (0)
d. I had a hard time getting the video to play	10 (8)	3 (6)	7 (10)
e. I liked watching the video	93 (79)	41 (82)	52 (76)
f. Other	10 (8)	5 (10)	5 (7)
Study enrollment intentions			
8. Did you decide to enroll in the OBOE study^d^?			
a. Yes	87 (67)	49 (89)	38 (51)
b. No	25 (19)	1 (2)	24 (32)
c. Not yet decided	17 (13)	5 (9)	12 (16)
8a. If you decided not to enroll or have not yet decided, what was the primary reason?			
a. Too much time or effort to complete the study visits or surveys	3 (8)	1 (17)	2 (6)
b. I do not trust that my information will be kept safe	2 (5)	0 (0)	2 (6)
c. I do not want to disclose any information, even if my name is not attached to it	0 (0)	0 (0)	0 (0)
d. I am not interested in participating in any research	5 (13)	0 (0)	5 (15)
e. I live too far away from the hospital or follow-up clinic	5 (13)	0 (0)	5 (15)
f. Financial burden to come back for the visits	1 (3)	0 (0)	1 (3)
g. I do not want my baby to have a brain MRI	6 (15)	0 (0)	6 (18)
h. Other	18 (45)	5 (83)	13 (38)

a OBOE: Outcomes of Babies With Opioid Exposure.

b MRI: magnetic resonance imaging.

c Percentages do not add to 100% because 3 respondents selected multiple responses.

d *P*<.001.

Survey responses did not differ significantly between caregivers of opioid-exposed infants and those of unexposed infants on all items except decision to enroll. Those in the opioid-exposed group were significantly more likely to report that they decided to enroll in the study compared to the unexposed group (n=49, 89% vs n=38, 51%; *P*<.001).

### Avatar Selection

Of 129 respondents who completed the consenter video survey, 1 (1%) was Asian, 1 (1%) was Black and Hispanic, 29 (22%) were Black and non-Hispanic, 69 (53%) White and non-Hispanic, and 29 (22%) were missing race. Among 81 respondents of known race who watched the video, 35 (43%) chose avatars to guide them through the video that matched their background. Participants who selected avatars consistent with their racial or ethnic background were more likely to report that they decided to enroll in the study (n= 29, 83% vs n=43, 57%; *P*=.01).

### Other Findings

An unanticipated benefit of the consenter video was its usefulness in informing new caregivers about the study when there were custody changes. As the longitudinal study progressed, coordinators indicated that the consenter video was helpful in providing information to new caregivers (eg, foster parents) about the study. We only asked caregivers to take the consenter survey at their infant’s enrollment into the study, so we do not have data to assess what new caregivers thought about the video in cases of custody changes.

## Discussion

### Principal Findings

Social distancing measures to mitigate the spread of COVID-19 placed limitations on face-to-face interactions between study staff and participants. The OBOE study addressed this challenge by developing an avatar-based consenter tool to supplement the consent process by creating an interactive video to explain study procedures while engaging a diverse cohort of study participants, particularly those from underrepresented populations who are most likely to have difficulty with traditional consent materials. Of the 129 caregivers who viewed the consenter video and took the survey, almost all (n=123, 95%) were able to correctly identify the purpose of the study, and nearly all felt that the video provided “just the right amount” of information and understood the consent information well enough to decide whether to enroll.

Participants’ most frequently cited strengths of the video were its ability to convey the importance of the study and its ability to explain the MRI and study procedures. This is an important finding given the lack of existing research assessing video-assisted consent for neuroimaging studies. In addition, when asked what they did not like about the video, 79% (n=93) of participants indicated they in fact liked watching the video, suggesting they were engaged in viewing the video-assisted consent. Compared with written consent forms, animation and visuals and such as those used in the consenter tool can better engage participants, potentially resulting in more informed research participants [22-24].

Participants who indicated that they did not like the consenter video cited technical issues (n=10, 8%) and length (n=5, 4%) as the primary reasons. Duration is a key element that affects engagement if the video length does not correlate with the complexity of the information conveyed. Researchers must try to avoid including excessive details, because doing so may result in informational overload thus minimizing the benefits of using a video [25,26]. Additionally, study staff must be adequately equipped to handle the technological needs associated with using digital tools. The OBOE study addressed the need for flexible viewing options by providing 3 ways for participants to view the video.

Given the potential for race-matched avatars to facilitate connections between participants and the study, we anticipated that participants would tend to choose the avatar that best matched their group identification. However, only 43% of participants whose race was known and who completed the consenter survey chose an avatar that aligned with their racial background. It is possible that participants chose avatars for reasons aside from self-representation, such as perceptions of avatars’ attractiveness or how authentic they felt an avatar looked. Lee [27] found that Black and female participants of a virtual video game were more likely to choose Black and female avatars (respectively) when assured that they were entering a high-diversity virtual world with other minority participants, but without this assurance, many Black participants chose White avatars. Lee [27] interpreted this finding using a social identity theory framework [28], explaining that virtual avatars can be used to facilitate self-expression and feelings of belonging, or they can create “escapes” for minority participants by allowing them to camouflage themselves as a member of the majority group. When virtual worlds are framed as high in diversity, participants tend toward the former, projecting their identity onto their avatar and customizing it accordingly. However, without this framing, participants may default to using ingrained cultural lenses and may “pass” under White avatars. Zimmermann et al [29], using the theories of transformed social interaction [30] and impression management [31], explain this “passing” behavior as people’s attempt to present an idealized representation of themselves in a given context.

Researchers must make active efforts to assure participants, through study framing and multimedia tool design, that the world they will enter as study participants is one that accepts them, includes them, and actively recognizes their autonomy to choose and consent, regardless of their background.

### Limitations

Specific activities that supported the launch of the consenter tool included receiving robust feedback from a diverse set of clinical site staff on the video illustrations, avatar images, and voice-overs to ensure the video was culturally relevant and met the needs of the caregivers approached for the study. However, a limitation is that we did not explicitly obtain feedback during video development from those with lived experience, such as caregivers of opioid-exposed infants. For example, Naeim et al [32] describe the implementation of electronic video consent in their precision health research after the formation of a community advisory board consisting of respected individuals from the participants’ home community who could speak of the communities’ perspectives. They solicited regular feedback from the community advisory board, ensuring that the video language was suitable for a lay population and spoke effectively to the study’s racially diverse sample.

### Conclusions

This interactive digital tool was helpful in informing prospective participants about the study and reinforcing why caregivers of unexposed infants were being approached. The video was a helpful resource for families to understand the study’s MRI procedures, because it provided a verbal description and included illustrations of a staff member preparing a swaddled, sleeping baby with protective earmuffs to enter the MRI scanner.

## Abbreviations

IRB: institutional review board
MRI: magnetic resonance imaging
OBOE: Outcomes of Babies With Opioid Exposure
REDCap: Research Electronic Data Capture
RTI: Research Triangle Institute
STROBE: Strengthening the Reporting of Observational Studies in Epidemiology
